# Establishing the Epidemiological Cut‐Off Value (ECOFF) for Cefquinome Against *Staphylococcus aureus* Using Standardised MIC Data

**DOI:** 10.1002/vms3.70995

**Published:** 2026-06-19

**Authors:** Ijaz Ahmad, Shakoor Ahmed, Muhammad Faizan, Shahab Ahmad Nawaz, Shabana Naz, Muhammad Israr, Rifat Ullah Khan, Antonella Perillo, Huda A. Alqahtani

**Affiliations:** ^1^ College of Veterinary Sciences The University of Agriculture Peshawar Pakistan; ^2^ Department of Zoology Government College University Faisalabad Pakistan; ^3^ Cardiff School of Pharmacy and Pharmaceutical Sciences Cardiff University Wales UK; ^4^ Department of Precision and Regenerative Medicine and and Ionic Area (DIMEPRE‐J) Veterinary medical sciences University of Bari ‘Aldo Moro’ Valenzano Italy; ^5^ Department of Zoology College of Science King Saud University Riyadh Saudi Arabia

**Keywords:** CO_WT_ and CO_PD_, *Staphylococcus aureus*, susceptibility breakpoints

## Abstract

*Staphylococcus aureus* is a major pathogen responsible for a wide range of infections in both animals and humans, and the increasing emergence of antimicrobial resistance highlights the need for effective surveillance tools. Cefquinome, a fourth‐generation cephalosporin, is widely used in veterinary medicine for the treatment of infections caused by Gram‐positive and Gram‐negative bacteria, including *S. aureus*. In this study, 110 *S. aureus* strains were isolated from cattle and subjected to minimum inhibitory concentration (MIC) determination using standardised agar dilution and microdilution methods. MIC values were obtained following 24 h incubation in 96‐well plates. The MIC distribution was analysed using goodness‐of‐fit testing and non‐linear least squares regression to establish the wild‐type cut‐off value (COWT). The MIC range for cefquinome against *S. aureus* was 0.03–2 µg/mL, and the epidemiological cut‐off value (ECV) was determined to be 2 µg/mL, encompassing 99.1% of the wild‐type population. An MIC value of 0.5 µg/mL covered 95% of the distribution, indicating its potential relevance for resistance monitoring. These findings provide a scientific basis for cefquinome susceptibility interpretation in cattle and support antimicrobial resistance surveillance. Further studies are warranted to develop population pharmacokinetic models for cefquinome in calves to optimise its therapeutic application.

## Graphical abstract

A total of 110 *Staphylococcus aureus* isolates from cattle were analysed to determine the epidemiological cut‐off value (ECOFF) for cefquinome. Minimum inhibitory concentrations (MICs) were determined using standardised agar dilution and broth microdilution methods following 24 h incubation. MIC distributions were evaluated using goodness‐of‐fit analysis and non‐linear least squares regression to define the wild‐type population. The MIC values ranged from 0.03 to 2 µg/mL, with 99.1% of isolates classified as wild type at an ECOFF of 2 µg/mL. An MIC of 0.5 µg/mL encompassed 95% of the population, indicating its relevance for resistance monitoring. These findings support standardised susceptibility interpretation of cefquinome in cattle and provide a framework for antimicrobial resistance surveillance and future PK/PD optimisation.

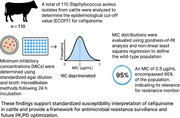

## 1 Introduction

Antimicrobial resistance (AMR) continues to escalate across major veterinary pathogens, posing a significant threat to animal health, food safety, and treatment efficacy. Increasing resistance has been documented in *Staphylococcus aureus*, *Streptococcus* spp., and *Enterococcus faecalis* isolated from dairy animals and livestock products (Nadi et al. 2024; Dongjing et al. 2024; Li et al. 2025; Bessembayeva et al. 2024). Similar trends have been reported in companion animals, where *Pseudomonas* spp. associated with otitis exhibit growing multidrug resistance (Mohammed et al. 2024). Methicillin‐resistant *S. aureus* (MRSA) has also been frequently detected in bovine mastitis, highlighting the expanding range of resistant strains in cattle populations (Abd El‐Razik et al. 2025).

In parallel, reports from diverse agricultural systems indicate increasing resistance in additional bacterial species, including *Streptococcus* spp. from yaks (Li et al. 2025) and multiple multidrug‐resistant pathogens associated with mastitis and wound infections (Ahsan et al. 2024; Rasool et al. 2024). Research focusing on pathogen isolation, MIC‐based susceptibility testing, and the evaluation of novel antimicrobial agents – such as selenium nanoparticles, silver oxide nanoparticles, plant extracts, and biologically derived compounds – continues to demonstrate the complexity of resistance mechanisms in veterinary pathogens (Ibrahim et al. 2024; Insumran et al. 2024; Kunu et al. 2025; Li et al. 2025; Ahsan et al. 2024). Beneficial microbes also show promise as alternative control options, as evidenced by the antagonistic activity of *Bacillus* spp. and *Streptomyces* metabolites against pathogenic bacteria (Fitriadi et al. 2024; Ryandini et al. 2024).

The ability of pathogens to form biofilms further complicates treatment outcomes. Biofilm formation by *S. aureus* and *Salmonella typhimurium* isolated from poultry houses contributes to enhanced persistence and antimicrobial tolerance (Laban et al. 2025). Similarly, *Staphylococcus* isolates from bovine mastitis exhibit high biofilm‐forming ability (Bessembayeva et al. 2024), while emerging evidence shows biofilm‐associated resistance in other veterinary pathogens such as uropathogenic *E. coli* (Gizinger et al. 2024). Innovative approaches, including bacteriophage‐based strategies, have shown potential for disrupting biofilms and limiting pathogen survival (Kinanti et al. 2024).

Given the rising prevalence of AMR and biofilm‐associated tolerance, the establishment of standardised antimicrobial susceptibility criteria is critical for improving therapeutic decision‐making. *Staphylococcus aureus*, a major pathogen causing mastitis in cattle, also poses risks to human health, being implicated in bacteremia, skin infections, soft tissue infections, and endocarditis (Park and Ronholm 2021; Tong et al. 2015; van der Vaart et al. 2022). Antimicrobial susceptibility testing plays an essential role in determining treatment effectiveness (Zaffiri et al. 2012). However, for many veterinary pathogens – including *S. aureus* in cattle – susceptibility breakpoints remain insufficiently defined (Rey et al. 2014; Kahlmeter and Turnidge 2023).

Breakpoint determination relies on key scientific components: MIC distributions, resistant strain selection, PK/PD relationships, and characterisation of wild‐type isolates. Statistical tools proposed by Meletiadis et al. (2012) and Kronvall (2010) are commonly applied, and breakpoints are categorised into wild‐type, clinical, and PK/PD types (Turnidge and Paterson 2007). When dosage regimen data are lacking, Monte Carlo simulations incorporating PK/PD indices and MIC distributions can be used to predict optimal clinical breakpoints (CLSI 2009; Aarestrup and Skov 2010). Although plasma drug levels are frequently employed for PK/PD modelling, drug concentrations at the infection site may more accurately reflect therapeutic outcomes (Barbour et al. 2010).

The objective of this study was to determine the minimum inhibitory concentrations (MICs) of cefquinome against 110 *Staphylococcus aureus* strains isolated from cattle and, based on these data, to establish the epidemiological cut‐off values (ECOFFs) through detailed MIC distribution analysis and identification of the wild‐type population. By generating these foundational parameters, the study aims to support the development of standardised antimicrobial susceptibility breakpoints for cefquinome, thereby improving the accuracy and reliability of therapeutic decision‐making in veterinary medicine.

## 2 Materials and Methods

A total of 111 clinical *Staphylococcus aureus* isolates were included in the present study. The isolates were obtained from cattle (*Bos taurus*) presenting clinical infections, and were recovered from milk samples collected from animals showing signs of mastitis at commercial dairy farms located in Peshawar, Pakistan during 2023. Following isolation, all strains were preserved in glycerol stocks at −70°C until further analysis. Prior to each experiment, the isolates were freshly subcultured on Chrom agar and Mueller–Hinton (MH) agar and incubated at 37°C for 24 h to ensure optimal growth and purity. Molecular confirmation of all isolates was performed using polymerase chain reaction (PCR) targeting the *S. aureus*‐specific nuc gene, as previously described by Karimzadeh and Ghassab (2022). The primers sequences are given below:

Nuc F GCGATTGATGGTGATACGGTT 3
Nuc R AGCCAAGCCTTGACGAACTAAAGC 3

### 2.1 Growth Curve Determination

Before MIC determination, the growth curves were checked in MH broth by counting the bacteria and also by spectrophotometer at wave length of 600. We also checked the growth stages of bacteria in both the shaker and incubator. From this study the different growth stages of bacteria were confirmed. The spectrophotometer and counting method were used. The samples were taken over 24 h to check the OD values and bacterial count (CFU/mL) from both samples, which were placed in a shaker incubator and a static incubator. This will helpful for MIC determination.

### 2.2 Minimum Inhibitory Concentration

Minimum inhibitory concentration was performed using the agar dilution method according to the CLSI M07‐A9 standard. A single bacterial colony was cultured in broth at 37°C, and the concentration was adjusted using a spectrophotometer to achieve a McFarland standard of 0.5. Bacterial cells in the logarithmic or lag phase were used for minimum inhibitory concentration (MIC) determination.

After inoculation, the agar plates were incubated at 37°C for 24 h. A 96‐well plate was prepared with a serial dilution of drug concentrations ranging from 0.01 µg/mL to 8 µg/mL. To ensure the reliability of the MIC results, *Staphylococcus aureus* (ATCC 12598) was used as quality control (QC) strain.

### 2.3 Definition of WT Distribution

The WT distribution included isolates without any acquired genotypic resistance mechanisms in a bacterium‐antibiotic combination (Turnidge et al. 2006; Kahlmeter and Turnidge 2023). And the CO_WT_ or ECV was the higher cut‐off of the distribution. Ideally, at least 95% WT isolates should be encompassed in the ECV.

#### 2.3.1 ECV

The ECV was calculated by following Turnidge method described (Turnidge et al. 2006). The WT distribution was conducted by normality test with sigma stat software v.3.5. Furthermore, to fit log_2_‐transformed MICs, nonlinear regression was used with Graphpad prism v.5.01. Finally, a WT distribution cut‐off was set by employed NORDIST and NORMINV functions.

## 3 Results

### 3.1 Confirmation of Bacteria

All 110 isolates of *Staphylococcus aureus* were confirmed using a combination of selective culture media, CHROMagar, and PCR. Initially, isolates were grown on selective media, where they exhibited characteristic colony morphology indicative of *S. aureus*. Further confirmation was performed using CHROMagar, where the isolates produced the expected colour reactions, reinforcing their identification.

To ensure molecular confirmation, PCR analysis was conducted using *nuc* gene specific primers, targeting specific *S. aureus nuc* genes. Gel electrophoresis results demonstrated the presence of the expected amplicon size of around 279 bp in all selected representative isolates (not shown). This multi‐step confirmation approach validated the identity of *S. aureus* isolates with high accuracy.

### 3.2 Growth Curve of *Staphylococcus aureus*

The growth curve of *Staphylococcus aureus* was analysed in Mueller–Hinton (MH) broth using both bacterial counting and spectrophotometric measurements at a wavelength of 600 nm. Growth stages were monitored under two different conditions: in a shaker and a static incubator, as shown in Table 1.

**TABLE 1:** Growth curve of *Staphylococcus aureus* under static and shaker incubator conditions measured by optical density (OD600) and colony‐forming units (CFU/mL).

A. Optical density (OD600)		
Time (h)	Static incubator	Shaker incubator
5	0.10	0.20
10	0.25	1.20
12	0.30	1.50
15	0.35	1.70
24	0.50	1.80

B. Colony forming units (CFU/mL)		
5	1.0 × 10^5^	5.0 × 10^5^
10	5.0 × 10^6^	5.0 × 10^7^
12	1.0 × 10^7^	8.0 × 10^7^
15	1.0 × 10^7^	1.0 × 10^8^
24	1.0 × 10^7^	1.0 × 10^8^

The study successfully confirmed the different growth phases of *S. aureus*, including lag, exponential, stationary, and decline phases. The combination of spectrophotometric readings and viable cell counts provided a reliable assessment of bacterial growth dynamics. This information is crucial for determining the optimal bacterial phase for minimum inhibitory concentration (MIC) testing. The bacterial concentrations were more than 10^6^ in shaker even after 2 h in shaker incubator; however the concentration of bacteria reached to 10^6^ within 3–4 h in static incubation. Meanwhile the OD value were checked and compared with counting method. The bacterial concentration at 10^6^ showed the OD value of 0.3.

### 3.3 Minimum Inhibitory Concentration

The minimum inhibitory concentration (MIC) for the reference strain *Staphylococcus aureus* (ATCC 29213) was determined to be 0.25 µg/mL. This value falls within the acceptable quality control (QC) range as recommended by the CLSI M31‐A3 guidelines, confirming the accuracy and reliability of the MIC testing method. The MIC values of different strains of *Staphylococcus auerus* are shown in Table 2. The inoculum preparation for MIC in broth culture was used. Inoculums density was further used by checking through spectrophotometer or Mc Farland value or 0.5. Usually 0.5 Mc Farland turbidity standard equal to colony count of 1–2 × 10^8^ CFU/mL, which will further 100‐fold diluted to make it 10^6^ CFU/mL, were used for MIC determination by micro dilution method.

**TABLE 2:** MIC distribution of *Staphylococcus aureus* strains.

MIC (µg/mL)	Bacterial strains
0.015	0
0.03125	1
0.0625	3
0.125	6
0.25	30
0.5	63
1	6
2	1
4	0
8	0

### 3.4 WT MIC Distribution and ECV

The distribution of wild type MIC for cefquinome against *S. aureus* ranged from 0.03 to 2 µg/mL. The cumulative log_2_ MIC results validated normal distribution which was confirmed through normality test (*p* = 0.200), even though there was continuous decrease of MIC at 0.5 µg/mL. Non‐linear regression as shown in Table 3 was used to obtain the optimum MIC range which is around 0.06–2 µg/mL. We used NORMINV function as shown in Table 4, to further adjust the optimum MIC range to 0.03–2 µg/mL. Similarly, NORMDIST function was used to calculate the probability of the higher and lower than high cut‐off value (0.18%) and low cut‐off value (0.07%), respectively. Therefore, as shown in Table 4, the ECV was expressed as 2 µg/mL and incorporated 99.1% of the wild type isolates.

**TABLE 3:** Optimal non‐linear least squares regression for pooled MICs (µg/mL) data.

Subset fitted	Number of isolates						Mean MIC (log_2_)				Standard deviation (log_2_)			
	True	Est.	Diff.	ASE	Est./ASE	95% CI^a^	Est.	ASE	Est./ASE	95% CI	Est.	ASE	Est./ASE	95% CI^a^
≦0.5	103	462	359	371.9	1.2	−1139, 2062	0.257	1.297	0.2	−5.323, 5.837	1.651	0.403	4.1	−0.000, 3.385
≦1	109	111	2	5.318	20.9	94, 128	−1.812	0.090	−20.1	−2.098, −1.527	0.625	0.128	4.9	0.217, 1.034
≦2	110	111	1	3.206	34.6	102, 119	−1.817	0.069	−26.3	−2.008, −1.625	0.617	0.102	6.0	0.334, 0.901

*Note*: Est., non‐linear regression estimate of value; Diff., estimate of N minus true N; ASE, asymptotic standard error; Est./ASE, estimate divided by asymptotic standard error.

^a^ 95% CI of estimate of value.

**TABLE 4:** The WT MIC range and ECV for cefquinome and *Staphylococcus aureus*.

MC range (µg/mL)	Optimum MIC range (µg/mL)	ECV (µg/mL)	ECV (%)
0.03–2	0.06–2	2	99.1

## 4 Discussion

In this study, we determined the minimum inhibitory concentration (MIC) of *Staphylococcus aureus* strains isolated from cattle. Antimicrobial susceptibility patterns can vary across different regions and may be influenced by factors such as sampling time, environmental conditions, and local antimicrobial usage (Espinel‐Ingroff et al. 2010; Smith et al. 2024). Broader microbial responses to changing environmental and management conditions have also been reported to influence pathogen dynamics and resistance trends in livestock systems (Anuoluwa et al. 2024). A total of 110 *S. aureus* strains were collected and analysed to assess their growth characteristics, species confirmation, and antimicrobial susceptibility, with a specific focus on establishing the epidemiological cut‐off value (ECV) for cefquinome.

Cefquinome is a fourth‐generation cephalosporin used for the treatment of severe bacterial infections, particularly when susceptibility testing indicates resistance to other antimicrobials (Ahmad et al. 2015). Monitoring resistance to critically important antimicrobials remains essential for safeguarding their therapeutic efficacy in veterinary medicine, particularly in the context of livestock disease transmission (Sarwar et al. 2025). The present study provides a comprehensive evaluation of the MIC distribution of cefquinome against *S. aureus*, contributing to a better understanding of resistance monitoring and epidemiological trends.

The growth curve analysis of *S. aureus* in Mueller–Hinton (MH) broth, assessed using both bacterial counting and spectrophotometric measurement at 600 nm, successfully delineated different bacterial growth stages under both shaking and static incubation conditions. Similar approaches integrating morphological, biochemical, and growth kinetics analyses have been shown to be reliable for characterising bacterial growth behaviour under controlled laboratory conditions (Guo et al. 2024; Xue et al. 2024)

The consistency between these methods confirms their reliability in assessing bacterial growth dynamics, which is crucial for optimising MIC determination. Standardising growth conditions is essential for ensuring reproducibility in antimicrobial susceptibility testing.

Molecular confirmation of all 110 *S. aureus* isolates through PCR further validated the bacterial identity, ensuring that only *S. aureus* isolates were included in the MIC determination. Molecular epidemiological approaches have been widely applied to enhance the accuracy of pathogen identification and resistance surveillance in bacterial populations (Dolhan et al. 2010), as also demonstrated in recent molecular epidemiology studies addressing infectious agents in animal and human health contexts (Ben Hadj Hassine et al. 2024).

The MIC determination for the reference strain *S. aureus* ATCC 29213 yielded a value of 0.25 µg/mL, which falls within the quality control (QC) ranges recommended by the CLSI M31‐A3 guidelines, confirming the reliability of the MIC testing protocol used in this study. The wild‐type (WT) MIC distribution for cefquinome against *S. aureus* ranged from 0.03 to 2 µg/mL, with a decline observed at an MIC of 0.5 µg/mL. The cumulative log2 MIC data followed a well‐defined normal distribution, as confirmed by the normality test (*p* = 0.200). Identifying this distribution pattern is critical for establishing ECVs, as it helps distinguish wild‐type bacterial populations from potentially resistant subpopulations (Turnidge et al. 2006; Feßler et al. 2023).

Using non‐linear regression, the optimal MIC range was initially determined as 0.06–2 µg/mL and was subsequently corrected to 0.03–2 µg/mL using the NORMINV function. Probability calculations performed using the NORMDIST function indicated that the probability of values exceeding the high cut‐off was 0.18%, while the probability of values below the low cut‐off was 0.07%. Based on these findings, the epidemiological cut‐off value (ECV) for cefquinome against *S. aureus* was established at 2 µg/mL, encompassing 99.1% of the wild‐type isolates.

The determination of an ECV is essential for distinguishing susceptible bacterial populations from those potentially harbouring acquired resistance mechanisms (Kahlmeter and Turnidge 2023; Gunnar Kahlmeter and John Turnidge 2024; Woo et al. 2022). The established ECV of 2 µg/mL serves as a valuable reference for future surveillance studies and antimicrobial resistance detection efforts. The well‐defined normal MIC distribution suggests minimal influence of acquired resistance within the studied population. However, continuous surveillance is recommended to monitor potential shifts in MIC distributions over time, which could indicate emerging resistance trends, particularly in the context of evolving livestock disease ecology (Sarwar et al. 2025).

## 5 Conclusion

In conclusion, this study provides a robust epidemiological framework for assessing cefquinome susceptibility in *S. aureus*. The integration of growth curve analysis, molecular confirmation, and statistical modelling ensures the reliability of the established ECV. These findings are expected to support antimicrobial stewardship efforts by improving resistance monitoring and guiding clinical decision‐making regarding cefquinome use against *S. aureus* infections.

## Author Contributions

**Shabana Naz**: writing – review and editing, writing – original draft. **Muhammad Faizan**: data curation. **Muhammad Israr**: funding acquisition. **I**: **Shahab Ahmad Nawaz**: software. **Ijaz Ahmad**: conceptualisation, methodology, validation, visualisation, software, investigation. **Shakoor Ahmed**: writing – original draft. **Antonella Perillo**: writing – review and editing. **Rifat Ullah Khan**: formal analysis. **Muhammad Israr**: author. **Huda A. Alqahtani**: Funding acquisition; Analysis

## Ethics Statement

The study was approved by the Ethical Committee of Faculty of Animal Husbandry & Veterinary Sciences, The University of Agriculture, Peshawar, Pakistan (Approval No. 12/FAH&VS/2021

## Conflicts of Interest

No potential conflict of interest was reported by authors

## AI

Artificial intelligence (ChatGPT 5) has partially been used for English language.

## Data Availability

The relevant data are provided in the paper. The data of the current experiment can be obtained from corresponding author when needed.

## References

[vms370995-bib-0001] Aarestrup, F. M. , and R. L. Skov . 2010. “Evaluation of Ceftiofur and Cefquinome for Phenotypic Detection of Methicillin Resistance in *Staphylococcus aureus* Using Disk Diffusion Testing and MIC‐Determinations.” Veterinary Microbiology 140: 176–179. 19643555 10.1016/j.vetmic.2009.07.005

[vms370995-bib-0002] Abd El‐Razik, K. A. , A. H. Soror , D. Sedky , E. A. Fouad , and A. A. Arafa . 2025. “Detection of Methicillin‐Resistant *Staphylococcus aureus* (MRSA) From Bovine Subclinical Mastitis Using Real‐Time PCR.” International Journal of Veterinary Science 14, no. 1: 188–195. 10.47278/journal.ijvs/2024.215.

[vms370995-bib-0003] Ahmad, I. , H. Hao , L. Huang , et al. 2015. “Integration of PK/PD for Dose Optimization of Cefquinome Against *Staphylococcus aureus* Causing Septicemia in Cattle.” Frontiers in Microbiology 6: 588. 26136730 10.3389/fmicb.2015.00588 PMC4470083

[vms370995-bib-0004] Ahsan, H. , M. Ayub , M. Gul , et al. 2024. “Efficacy of Silver Oxide Nanoparticles Against Multi‐Drug Resistant *Pseudomonas aeruginosa* and Methicillin‐Resistant *Staphylococcus aureus* in Burn Wound Infections.” Asian Journal of Agriculture and Biology 2024, no. 4: 2024096. 10.35495/ajab.2024.096.

[vms370995-bib-0005] Anuoluwa, I. A. , E. A. Ekundayo , O. O. Bello , Y. D. Oluwafemi , I. A. Adesina , and B. E. Bolajoko . 2024. “Microbial Responses to Shifting Climate Patterns.” Agrobiological Records 17: 42–57. 10.47278/journal.abr/2024.021.

[vms370995-bib-0006] Barbour, A. , F. Scaglione , and H. Derendorf . 2010. “Class‐Dependent Relevance of Tissue Distribution in the Interpretation of Anti‐Infective Pharmacokinetic/Pharmacodynamics Indices.” International Journal of Antimicrobial agents 35: 431–438. 20219329 10.1016/j.ijantimicag.2010.01.023

[vms370995-bib-0007] Ben Hadj Hassine, A. , M. Marzouk , J. Saad , J. Boukadida , and M. Drancourt . 2024. “Molecular Epidemiology of *Mycobacterium Tuberculosis* Complex in the Center of Tunisia (2008–2010 and 2014–2017).” Agrobiological Records 17: 69–74. 10.47278/journal.abr/2024.024.

[vms370995-bib-0008] Bessembayeva, L. , Z. Kirkimbayeva , S. Yergaliyeva , A. Kumisbek , and F. Bakiyeva . 2024. “Investigation of the Antibiotic Resistance and Biofilm‐forming Ability of *Staphylococcus* Species From Bovine Mastitis Cases.” International Journal of Veterinary Science 13, no. 6: 853–861. 10.47278/journal.ijvs/2024.172.

[vms370995-bib-0009] CLSI . 2009. “Development of In Vitro Susceptibility Testing Criteria and Quality Control Parameters for Veterinary Antimicrobial Agents; Approved Guideline‐Third Edition, Document M37‐A3.” Clinical and Laboratory Standard Institute 28, no. 7: 11.

[vms370995-bib-0010] Dolhan, A. , A. Jelinska , and M. Bebenek . 2014. “Stability of Ceftiofur Sodium and Cefquinome Sulphate in Intravenous Solutions.” The Scientific World Journal 2014: 583461. 10.1155/2014/583461. 25025091 PMC4065735

[vms370995-bib-0011] Dongjing, W. , S. Zhonghua , Y. Zhenjie , and M. H. Almutairi . 2024. “Isolation, Identification, and Biological Characteristics of Pathogenic *Enterococcus faecalis* From Tibetan Sheep.” Asian Journal of Agriculture and Biology 2024, no. 3: 2023327. 10.35495/ajab.2023.327.

[vms370995-bib-0012] Espinel‐Ingroff, A. , D. J. Diekema , A. Fothergill , et al. 2010. “Wild‐Type MIC Distributions and Epidemiological Cutoff Values for the Triazoles and Six *Aspergillus* spp. For the CLSI Broth Microdilution Method (M38‐A2 Document).” Journal of clinical Microbiology 48: 3251–3257. 20592159 10.1128/JCM.00536-10 PMC2937688

[vms370995-bib-0013] Feßler, A. T. , Y. Wang , C. R. Burbick , et al. 2023. “Antimicrobial Susceptibility Testing in Veterinary Medicine: Performance, Interpretation of Results, Best Practices and Pitfalls.” One Health Advances 1: 26.

[vms370995-bib-0014] Fitriadi, R. , A. Sabdaningsih , S. B. Prayitno , P. H. T. Soedibya , Sarjito , and Subagiyo,. 2024. “ *Bacillus* spp. Isolated From White Shrimp *Fenneropenaeus merguiensis* and Antagonistic Activity Against Vibrio Pathogens.” International Journal of Agriculture and Biosciences 13, no. 3: 410–418. 10.47278/journal.ijab/2024.137.

[vms370995-bib-0015] Gizinger, O. , V. Radzinskiy , Y. Sorokin , et al. 2024. “The Effects of Interleukin‐2 Substances on Biofilm Formation in Multidrug‐Resistant Uropathogenic *Escherichia coli* in Women With Previous Reproductive Losses.” International Journal of Agriculture and Biosciences 13, no. 4: 753–762. 10.47278/journal.ijab/2024.185.

[vms370995-bib-0016] Guo, W. , T. Ahmad , D. Li , and Y. Liu . 2024. “Morphological and Biochemical Characterization of *Novosphingobium* Species and Their Optimal Growth Kinetics.” Agrobiological Records 15: 68–74. 10.47278/journal.abr/2024.001.

[vms370995-bib-0017] Ibrahim, E. S. , A. M. Abdalhamed , A. A. Arafa , et al. 2024. “In Vitro and In Vivo Antibacterial and Antibiofilm Efficacy of Selenium Nanoparticles Against *Staphylococcus aureus* .” International Journal of Veterinary Science 13, no. 4: 490–500. 10.47278/journal.ijvs/2023.115.

[vms370995-bib-0018] Insumran, Y. , J. Sriwongsa , K. Kaewprom , W. Khonsuntia , and M. Intrakhamhaeng . 2024. “Exploring the Antimicrobial Potential of *Spirogyra neglecta* Against Mastitis‐Inducing Pathogens.” International Journal of Veterinary Science 13, no. 5: 700–706. 10.47278/journal.ijvs/2024.156.

[vms370995-bib-0019] Kahlmeter, G. , and J. Turnidge . 2023. “The Determination of Epidemiological Cut‐Off Values Requires a Systematic and Joint Approach Based on Quality Controlled, Non‐Truncated Minimum Inhibitory Concentration Series.” European Respiratory Journal 61, no. 5: 2202259. 37147008 10.1183/13993003.02259-2022

[vms370995-bib-0020] Kahlmeter, G. , and J. Turnidge . 2023. “Wild‐Type Distributions of Minimum Inhibitory Concentrations and Epidemiological Cut‐Off Values – Laboratory and Clinical Utility.” Clinical Microbiology Reviews 36: e00100–e00122. 38038445 10.1128/cmr.00100-22 PMC10732016

[vms370995-bib-0021] Kahlmeter, G. , and J. Turnidge . 2024. “How To: ECOFFs – The Why, the How, and the Don'ts of EUCAST Epidemiological Cutoff Values.” Clinical Microbiology and Infection 28, no. 7: 952–954. 10.1016/j.cmi.2022.02.024 35218980

[vms370995-bib-0022] Karimzadeh, R. , and R. K. Ghassab . 2022. “Identification of Nuc Nuclease and Sea Enterotoxin Genes in *Staphylococcus aureus* Isolates From Nasal Mucosa of Burn Hospital Staff: A Cross‐Sectional Study.” New Microbes and New Infections 47: 100992. ISSN 2052–2975. 35800028 10.1016/j.nmni.2022.100992 PMC9253491

[vms370995-bib-0023] Kinanti, A. S. , A. A. Prihanto , Y. D. Jatmiko , R. Kobun , and W. X. L. Felicia . 2024. “Harnessing Bacteriophages: A Promising Approach to Combat Foodborne Pathogen Biofilms.” International Journal of Agriculture and Biosciences 13, no. 4: 656–668. 10.47278/journal.ijab/2024.172.

[vms370995-bib-0024] Kronvall, G. 2010. “Normalized Resistance Interpretation as a Tool for Establishing Epidemiological MIC Susceptibility Breakpoints.” Journal of Clinical Microbiology 48: 4445–4452. 20926714 10.1128/JCM.01101-10 PMC3008453

[vms370995-bib-0025] Kunu, W. , R. Meekrasae , P. Kongpheng , J. Samart , and S. Patathananone . 2025. “Effectiveness of *Spondias Pinnata* Fruit Extracts Against Antibiotic‐Resistant Mastitis‐Causing Bacteria.” International Journal of Veterinary Science 14, no. 1: 196–203. 10.47278/journal.ijvs/2024.225.

[vms370995-bib-0026] Laban, S. E. , A. A. Arafa , E. S. Ibrahim , F. E. Eman , and H. S. Khalefa . 2025. “Dry Biofilm Formation by *Salmonella Typhimurium* and *Staphylococcus aureus* Isolated From Poultry Houses.” International Journal of Veterinary Science 14, no. 1: 25–31. 10.47278/journal.ijvs/2024.197.

[vms370995-bib-0027] Li, L. , B. Zhang , D. Li , et al. 2025. “Evaluation of Antibacterial, Anti‐inflammatory, and Anti‐Allergy Properties of *Isodonis japonicus* Extract.” Asian Journal of Agriculture and Biology 2025: 2024154. 10.35495/ajab.2024.154.

[vms370995-bib-0028] Li, Z. , J. Huang , Z. Bai , et al. 2025. “The Epidemiology of Drug Resistance in *Streptococcus* Species Isolated From Yaks in Tibet.” Asian Journal of Agriculture and Biology 2025: 2024099. 10.35495/ajab.2024.099.

[vms370995-bib-0030] Meletiadis, J. , E. Mavridou , W. J. G. Melchers , J. W. Mouton , and P. E. Verweij . 2012. “Epidemiological Cutoff Values for Azoles and *Aspergillus fumigatus* Based on a Novel Mathematical Approach Incorporating cyp51A Sequence Analysis.” Antimicrobial Agents and Chemotherapy 56: 2524–2529. 22330922 10.1128/AAC.05959-11 PMC3346643

[vms370995-bib-0031] Mohammed, B. Q. , A. H. Abdullah , and A. R. Rayshan . 2024. “ *Pseudomonas* That Causes Otitis in Dogs: An Increasing Opposition.” International Journal of Agriculture and Biosciences 13, no. 1: 59–64. 10.47278/journal.ijab/2024.087.

[vms370995-bib-0032] Nadi, W. G. , L. I. Ahmed , A. A. N. Awad , and E. M. Taher . 2024. “Occurrence, Antimicrobial Resistance, and Virulence of *Staphylococcus aureus* Isolated From Dairy Products.” International Journal of Veterinary Science 13, no. 2: 218–225. 10.47278/journal.ijvs/2023.079.

[vms370995-bib-0033] Park, S. , and J. Ronholm . 2021. “ *Staphylococcus aureus* in Agriculture: Lessons in Evolution From a Multispecies Pathogen.” Clinical Microbiology Reviews 34: e00182–e00220. 10.1128/cmr.00182-20. 33568553 PMC7950364

[vms370995-bib-0034] Rasool, M. , M. H. Rasool , M. Khurshid , and B. Aslam . 2024. “Biogenic Synthesis and Characterization of Silver Nanoparticles: Exploring Antioxidant and Anti‐Inflammatory Activities and Assessing Antimicrobial Potential Against Multidrug‐Resistant Bacteria.” Asian Journal of Agriculture and Biology 2024, no. 3: 2023364. 10.35495/ajab.2023.364.

[vms370995-bib-0035] Rey, J. F , C. M. Laffont , S. Croubels , et al. 2014. “Use of Monte Carlo Simulation to Determine Pharmacodynamic Cutoffs of Amoxicillin to Establish a Breakpoint for Antimicrobial Susceptibility Testing in Pigs.” American Journal of Veterinary Research 75, no. 2: 124–131. 24471748 10.2460/ajvr.75.2.124

[vms370995-bib-0037] Ryandini, D. , S. Ma'arif , A. Hidayat , et al. 2024. “Antibacterial, Antiaging, and Antiangiogenic Activity of *Streptomyces sp*. SAE4034 Extract From Mangrove Sediment.” International Journal of Agriculture and Biosciences 13, no. 4: 574–581. 10.47278/journal.ijab/2024.160.

[vms370995-bib-0038] Sarwar, M. Z. , Z. A. Nomi , M. Awais , et al. 2025. “Effect of Climate Change on Transmission of Livestock Diseases.” Agrobiological Records 19: 1–11. 10.47278/journal.abr/2025.001.

[vms370995-bib-0039] Smith, P. , A. Joseph , C. Baker‐Austin , et al. 2024. “Epidemiological Cut‐Off Values for Vibrio Parahaemolyticus Calculated From Minimal Inhibitory Concentration Data Generated at 35 and 28°C.” Diseases of Aquatic Organisms 160: 127–134. 39665310 10.3354/dao03831

[vms370995-bib-0040] Tong, S. Y. C. , J. S. Davis , E. Eichenberger , T. L. Holland , and V. G. Fowler . 2015. “ *Staphylococcus aureus* Infections: Epidemiology, Pathophysiology, Clinical Manifestations, and Management.” Clinical Microbiology Reviews 28, no. 3: 603–661. 10.1128/CMR.00134-14. 26016486 PMC4451395

[vms370995-bib-0041] Turnidge, J. , G. Kahlmeter , and G. Kronvall . 2006. “Statistical Characterization of Bacterial Wild‐Type MIC Value Distributions and the Determination of Epidemiological Cut‐Off Values.” Clinical Microbiology and Infection 12: 418–425. 16643517 10.1111/j.1469-0691.2006.01377.x

[vms370995-bib-0042] Turnidge, J. , and D. L. Paterson . 2007. “Setting and Revising Antibacterial Susceptibility Breakpoints.” Clinical Microbiology Reviews 20: 391–408. 17630331 10.1128/CMR.00047-06 PMC1932754

[vms370995-bib-0043] van der Vaart, T. W. , J. M. Prins , R. Soetekouw , et al. 2022. “Prediction Rules for Ruling out Endocarditis in Patients With *Staphylococcus aureus* Bacteremia.” Clinical Infectious Diseases 74, no. 8: 1442–1449. 34272564 10.1093/cid/ciab632 PMC9049276

[vms370995-bib-0046] Woo, S.‐J.i , M.‐S. Kim , M.‐G. Jeong , M.i‐Y. Do , S.‐D. Hwang , and W.‐J. Kim . 2022. “Establishment of Epidemiological Cut‐Off Values and the Distribution of Resistance Genes in *Aeromonas Hydrophila* and *Aeromonas Veronii* Isolated From Aquatic Animals.” Antibiotics 11, no. 3: 343. 35326806 10.3390/antibiotics11030343 PMC8944483

[vms370995-bib-0048] Xue, Q. , T. Ahmad , and Y. Liu . 2024. “Morphological, Physiological and Biochemical Characterization of *Pseudoxanthomonas* Species and Its Optimal Growth Kinetics.” Agrobiological Records 16: 41–48. 10.47278/journal.abr/2024.010.

[vms370995-bib-0049] Zaffiri, L. , J. Gardner , and L. H. Toledo‐Pereyra . 2012. “History of Antibiotics: From Salvarsan to Cephalosporins.” Journal of Investigative Surgery 25, no. 2: 67–77. 22439833 10.3109/08941939.2012.664099

